# Genomic and phenotypic diversities among *Bacillus cereus* isolates from cosmetics

**DOI:** 10.1186/s12866-025-04311-x

**Published:** 2025-12-19

**Authors:** Nadine Yossa, Roma Adu-Osei, Travis Canida, Gopal Gopinath, Rachel Binet

**Affiliations:** 1 Food and Drug Administration/Human Foods Program/Office of Laboratory Operations and Applied Science, Office of Applied Microbiology and Technology, College Park, MD 20740 USA; 2 Oak Ridge Institute for Science and Education, Oak Ridge, TN 37831 USA; 3 FDA/HFP/Office of Surveillance Strategy and Risk Prioritization, Division of Surveillance and Data Integration/Surveillance Design Branch, College Park, MD 20740 USA

**Keywords:** *B. cereus* Cosmetic Isolates, GalaxyTrakr, BV-BRC, VITEK®2, Phenotype Microarrays™, Genotypic, Phenotypic, Sensitivity, Resistance

## Abstract

**Background:**

As *Bacillus cereus* species can negatively affect the safety and quality of both food and non-food products, it would be useful to describe among the selected strains traits supporting survival in specific matrices. We recently characterized the genome of 3 A ES, a *B. cereus* isolated from eye shadow, revealing multiple genes predicted to support the adaptation of this strain to harsh environmental conditions. Here, we compared the genotypic and phenotypic relative sensitivity/ resistance of *B. cereus* 3 A ES with two additional *B. cereus* cosmetic isolates: an eye shadow isolate, 7 C ES, a baby wipe isolate, 1 L BW, as well as clinical strain American Type Culture Collection (ATCC) 49,063, an emetic strain from unknown origin F60006, the ATCC 14,579 reference type strain, and a non-*cereus* strain, *B. pumilus* ATCC 7061. An additional eye shadow isolate, 8 A ES, was only tested phenotypically.

**Results:**

BTyper, the *B. cereus* subtyping tool within the FDA HFP GalaxyTrakr bioinformatic platform, separated the strains in three phylogenetic groups, with *B. pumilus* as an outlier. Four strains were identified as *B. cereus* sensu stricto (s.s.), group IV: 3 A ES, ATCC strains 14,579 and 49,063, with 7 C ES additionally identified as *B. cereus* s.s. biovar Thuringiensis. F60006 was recognized as *B. mosaicus* subsp. *cereus* Emeticus s.s., in group III. Isolate 1 L BW was identified as *B. cytotoxicus*, group VII. Analyses of these genomic sequences using the Pathosystems Resource Integration Center (BV-BRC - PATRIC) revealed genes predicting resistance for diverse antibiotic groups and biocides. *B. cereus* strains displayed resistance to most of the compounds tested with Vitek^®^ 2 Compact system and phenotype microarrays™. No differences were found between the overall average growth that correlates with the respiration response between the isolates from cosmetics and non-cosmetics (*p* = 0.56). However, there was a significant difference (*p* < 0.001) in the overall sensitivity/resistance phenotypes of the strains: the non-cereus ATCC 7061 was the least resistant strain and 3 A ES, the most resistant.

**Conclusion:**

Although these *B. cereus* strains exhibited a range of genotypic and phenotypic profiles, there were no traits distinguishing isolates from cosmetics from the isolates from non-cosmetics tested. Further, antimicrobial resistance was not only dependent on the arsenal carried by a given strain but also on the concentration of each antibiotic.

**Supplementary Information:**

The online version contains supplementary material available at 10.1186/s12866-025-04311-x.

## Introduction

The *Bacillus cereus* group, also known as *B*. *cereus* sensu lato (s.l.), are rod-shaped Gram-positive spore-forming bacteria, comprised of at least twenty-two closely related species [1]. Members of this group are known for their resiliency under a wide variety of environmental challenges such as heat, desiccation, radiation, and a variety of chemicals used for cleansing and antibiotic purposes [2]. Strains are capable of persisting in soil, rhizosphere, plants, sea water, food, processed foods, and cosmetic products – whether or not preservatives were used to prevent such persistence [3, 4] – and even the surface of spacecraft [5, 6]. Some members of this group have been associated with food poisoning [7, 8], eye damage, and other human infections [9, 10], while other strains have been useful for the fermentation of foods and insect control [11].

As many researchers have found, the close genetic proximity of *B. cereus* strains makes differentiation among them challenging [11, 12]. Currently, members of the *B. cereus* group are classified based on sequencing the *pan*C housekeeping gene, which encodes for the pantoate-beta alanine ligase C. This system classes *B. cereus* in seven phylogenetic Groups (I-VII), which overlap with their range of preferred growth temperatures, although it is important to note factors such as mutation and adaptation to low temperatures can result in strains typically recognized as separate species to be found together within these seven groups [13, 14]. Group I-VII was later adjusted to a I-VIII group panC/genomospecies, which reduce overlapping and allow a better delineation between genomes by employing a combination of virulence gene-based typing, multilocus sequence typing, *panC* clade typing, and *rpoB* allelic typing to reconcile genomic definitions of bacterial species with clinical and industrial phenotypes, and the tool uses the standardized *B. cereus s.l.* genomospecies/subspecies/biovar framework [15]. However, regardless of the classification methods used, taxonomic challenges still exist. The broad range of temperature tolerances may help explain how *B. cereus* group members can inhabit such a broad range of environmental niches, and species definitions may help species- phenotype compatibility, especially when horizontal gene transfer is responsible for a phenotype [13].

However, less is known about whether particular genes or products contribute to the persistence of *B. cereus* in specific matrices, particularly cosmetics or foods. Identifying genomic features that might distinguish *B. cereus* strains found in cosmetics from strains obtained from other matrices, such as foods, would be extremely useful, especially if paired with phenotypic assays to confirm predicted sensitivity/resistance profiles. Here we compare genotypic and phenotypic relative sensitivity/resistance of five cosmetic isolates: three obtained from eye shadow (3 A ES [16], 7 C ES, 8 A ES), one obtained from baby wipes (1 L BW) and one known to survive for long periods of time in cosmetic creams, (F60006) [17, 18], . Two strains not associated with cosmetics, the food testing reference strain ATCC 14579 and the clinical outbreak strain ATCC 49063, were used as the comparators, with the non-*cereus* ATCC 7061 *B. pumilus* closely related to *B. pumilus* SAFR-032 isolated from spacecraft and assembly-facility surfaces that displays unusual resistance to elevated temperatures [5, 6], used as the control. Phenotypic testing was performed using both the Vitek 2 System ((bioMerieux, Durham, NC) and Phenotype microarrays™ (PM) (Biolog, Hayward, CA, US), which evaluate microbial growth in the presence of multiple antimicrobial compounds (sensitivity/resistance).

## Materials and methods

### Bacterial strains

Bacterial cultures were maintained at ^−^80 °C in 20% glycerol, then each strain was aseptically sub-cultured in nutrient broth (NB) (DifcoTM, Franklin Lakes, NJ) for 24 h at 35.0 °C and stored at 4 °C for use (Table 1).

**Table 1:** Bacterial strains and sources

Bacteria	Strain	Source	Detail
*B. cereus*	3 A ES	Eye Shadow – cosmetics	Previously characterized (16)
*B. cereus*	7 C ES	Eye Shadow – cosmetics	FDA San Francisco Laboratory
*B. cereus*	8 A ES	Eye Shadow – cosmetics	Used for phenotypic comparisons in this study
*B. cereus*	1 L BW	Baby wipes – cosmetics	FDA San Francisco Laboratory
*B. cereus*	F60006	Unknown	Demonstrated long term survival in cosmetic creams (17)
*B. cereus*	49,063	ATCC	Clinical outbreak strain
*B. cereus*	14,579	ATCC	Food testing reference strain
*B. pumilus*	7061	ATCC	Closely related to *B. pumilus* SAFR-032 (6)

### Genomic characterization of selected strains

After overnight incubation at 35 °C in NB, genomic DNA was extracted using the DNeasy Blood and Tissue Kit (Qiagen Inc, Valencia, CA). DNA concentrations were measured using a Qubit 3.0 fluorometer (Life Technologies, MD). Sequencing libraries were prepared following Nextera XT protocols and sequenced on the Illumina MiSeq desktop sequencer (Illumina, San Diego, CA) according to the manual. Sequences were trimmed and de novo assembled with the Unicycler v0.4.8 sets on default parameters. Genomic sequence data of the seven strains which passed quality assessments were used for further characterization and phylogenetic analyses. Data availability for the strains used is shown in Table 2.

**Table 2:** Strain data availability

SUBID	BioProject	BioSample	SRA	Organism
SUB14244836	PRJNA574468	SAMN38019395	–	*B. cereus* 1 L BW
–	–	SAMN38019394	SRS20346157	*B. cereus* 7 C ES
–	–	SAMN17257548	SRR13386521	*B. cereus* 3 A ES

The Resistant Gene Identifier (RGI) tool in GalaxyTrakr Version 2.0.3 was used to identify antibiotic resistance genes from either protein or nucleotide data, based on homology and single nucleotide polymorphism (SNP) models drawn from the comprehensive antibiotic resistance database (CARD). To classify our *B. cereus* group isolates, we used BTyper, which combines virulence gene-based typing, multi-locus sequence typing (MLST), *panC* clade typing, and *rpoB* allelic typing [12].

### Phylogenetic analyses

Bacterial and Viral Bioinformatics Resource Center (BV-BRC) provides and allows researchers to use reference databases and other representative genomes and includes these in phylogenetic analysis as part of their Comprehensive Genome Analysis reports. The phylogenetic tree was generated by the Codon tree pipeline with a total of twenty-five genomes including 3 A ES, 7 C ES, IL-BW, F60006, ATCC 49,063, and ATCC 14,579 in BV-BRC [19]. It uses the amino acid and nucleotide sequences from a defined number of the BV-BRC global protein families (PGFarms) [20], which are picked randomly, to build an alignment, and then generate a tree based on the differences within those selected sequences. Both the protein (amino acid) and gene (nucleotide sequences) are used for each of the selected genes from the PGFams. Protein sequences are aligned using MUSCLE and BioPython were used for the alignment of protein and the nucleotide coding gene sequences [21, 22]. Then, all concatenated proteins and nucleotides alignments are written to PHYLIP, and a partitions file for RaxML [23] is generated. Bootstrap support values were 100 [24]. The genome quality analysis is automatically performed and included in the genome annotation report. Completeness, contamination, coarse consistency, and fine consistency measure the quality of the genome. The genomes that meet the quality analysis were used for the study.

### BV-BRC comparative systems analyses

The pangenomes of 3 A ES, ATCC 49,063 and 14,579 were compared using the Protein Family Sorter part of the Comparative Systems Service in BV-BRC [20, 25, 26]. The resulting core genomes, those protein families conserved across the genomes and accessory genomes, as well as the protein families that are conserved in a subset of the selected genomes or that match a specified function were used to construct a three-part Venn diagram, to better visualize which accessory proteins distinguish 3 A ES from the other strains.

### Phenotype Vitek^®^ 2 sensitivity/resistance tests

The Vitek^®^ 2 system (bioMerieux, Durham, NC) was used to compare the sensitivity/resistance of the strains using the BCL card for Gram-positive microorganisms of the family Bacillaceae (bioMerieux). The BCL card contains 46 substrates which assess carbon source utilization, enzymatic activities, inhibition by 6.5% NaCl, and resistance to three antibiotics: kanamycin, oleandomycin and polymyxin B [27].

### Phenotype Microarray™-Biolog (PM) tests

To assess the sensitivity/resistance phenotypes of the eight strains against 240 different antimicrobials and biochemical stressors, including 44 compounds used in the pharmaceutical and cosmetic industries, we used the 10 Phenotype Microarray plates for assessing bacterial sensitivity: PM 11 C through PM 20B (Biolog). First, eight strains (3 A ES, 7 C ES, 8 A ES, 1 L BW, ATCC 14579, F60006, ATCC 49063, and ATCC 7061) were grown overnight at 33 °C on BUG agar (Biolog). Cells were picked with a sterile cotton swab and suspended in 20 ml inoculation fluid (IF-0, Biolog). Cell density was adjusted to 81% transmittance (T) on a Biolog turbidimeter, and cell suspension (9.68 ml) was added to inoculating fluid (122.32 ml).

Each 96-well PM plate contains 24 different antimicrobials at four unknown, but increasing, concentrations in four separate wells. These wells were inoculated with 100 µl cell suspensions, then incubated at 33 °C in an Omnilog Reader (Biolog). Tetrazolium violet is used to report active metabolism, as the reduction of this dye causes the formation of a purple color that is recorded by a charge-coupled device camera every 15 min. This provides quantitative and kinetic information about the effects of these antimicrobials on the cells in each well [28]. Readings were recorded for 48 h, and data were analyzed with Omnilog-PM software (release OM_PM_109M, Biolog), which generated a time-course curve for tetrazolium color formation. Any well that does not exhibit the purple color (a “clear” well) indicates no active metabolism; each strain is scored by the number of clear wells for each challenge compound. Thus, a strain that grows in all four concentrations has zero clear wells, and is scored 0, indicating high resistance to that challenge. A score of 1 is considered resistant, 2 = partially resistant, 3 = minimally resistant and 4 means that strain exhibits no resistance.

Each strain was analyzed in duplicate, and the average was obtained. In total, 1920 different results were collected, corresponding to eight strains tested against 240 different antimicrobial compounds.

### Statistical analysis

Data analysis was performed using a principal components analysis (PCA), widely used for PM experiments [11, 29, 30], as it reduces the dimensionality of a given dataset while still retaining as much variance as possible. To analyze the average growth/respiration of each strain in each PM well, the PCA used only the first two principal components. To determine whether a certain strain showed resistance to a chemical in the PM wells, an intercept only linear model was fitted on the first order difference of the strain’s growth in each PM well, then tested to determine whether that intercept was statistically different from zero at the 0.05 significance level. If that standard was met, it indicated the exponential growth of the strain in that PM well, and implied that the strain showed resistance to that chemical challenge. All analyses were performed using R version 4.1.2 [31].

## Results and discussion

*B. cereus* group has been associated not only with food poisoning and gastrointestinal diseases but also with non-gastrointestinal diseases [32]. Although the impacts of *B. cereus* in cosmetics are rare [33], there have been reports of recalled baby wipes contaminated with *B. cereus* group member [34]. Our results present a base study of the compounds that could be used in cosmetics to prevent *B. cereus* s.l. from growing in the products (See Supplemental Table of products used in cosmetics).

### Molecular characterization of selected strains

The comprehensive genome analysis provided by BV-BRC revealed that the assembled draft genomes were of good quality, except for strain 8 A ES that was contaminated; therefore, that strain was only used for phenotypic analyses. As the characteristics of *B*. *cereus* 3 A ES genome had been published previously [16], it served as the reference genome for remaining *B. cereus* cosmetics strains in this study.

The CFSAN GalaxyTrakr BTyper (Table 3) classified the assembled genomes of seven strains into 3 groups: Three strains, one cosmetic strain, 3 A ES, and two type strains, ATCC 14,579 and ATCC 49,063, belonged to Group IV and were identified as *B. cereus* sensu stricto (s.s.). Although cosmetic strain 7 C ES was also assigned to Group IV, the presence of genes coding for the insecticidal crystal proteins identified it as *B. cereus* s.s. biovar Thuringiensis. The strain F60006 belonged to Group III and was identified as *B. mosaicus* subsp. *cereus* Emeticus s.s. The last cosmetic strain, 1 L BW, belonged to Group VII and was identified as *B. cytotoxicus*. As ATCC 7061 is a *B. pumilus* strain, it did not fit into any of the *B. cereus* groups.

**Table 3:** BTyper analysis: selected pathogenicity factors and proposed taxonomy

Strains	Predicted virulence factors						PanC Group	Taxon Names
	Emetic toxin	Non-hemolysin	Hemolysin	cytotoxin	Sphingo- myelinase	Crystal toxin		
3 A ES	No	NheABC	HblABCD	cytK-2	*sph*	No	IV	*B. cereus* s. s.
7 C ES	No	NheABC	HblABCD	cytK-2	*sph*	20; *cry*	IV	*B. cereus* s. s. biovar Thuringiensis
8 A ES	No	NheAB	HblABC	cytK-2	*sph*	No	IV	*B. cereus* s. s.
1 L BW	No	NheABC	NA	cytK-1	*sph*	No	VII	*B. cytotoxicus*
ATCC 14,579	No	NheABC	HblABCD	cytK-2	*sph*	No	IV	*B. cereus* s. s.
F60006	*ces*ABCD	NheABC	*NA*	NA	*sph*	No	III	*B. mosaicus subsp. cereus* Emeticus s. s.
ATCC 49,063	No	NheAB	HblABD	cytK-2	No	No	IV	*B. cereus* s. s.
ATCC 7061	No	No	No	No	No	No	No	No

NHE: non-hemolytic enterotoxin; HBL: hemolytic enterotoxin; *cyt*K: cytotoxin K; *sph*: sphingomyelinase; *cry*: crystal protein; *ces*: emetic toxin

### Predicted toxin and antimicrobial resistance genes

The BTyper analysis (Table 3) predicted genes coding for the tripartite non hemolytic toxin, *nhe*ABC, the hemolysin *hbl*ABCD, and the cytotoxin, *cyt*K-2, associated with diarrheal diseases, would be found in three strains: 3 A ES, 7 C ES and ATCC 14,579. The clinical outbreak strain ATCC 49,063 did not carry either *nhe*C or *hbl*C, it did carry *cyt*K-2. Strain 1 L BW harbored the *nhe*ABC and the *cyt*K-1 genes, while F60006 harbored both *nhe*ABC and the gene cluster *ces*ABCD, which codes for the heat-stable emetic toxin cereulide.

Genes for antibiotic resistance were predicted by the RGI tool in GalaxyTrakr (Table 4). Genes for resistance to fosfomycin, *fos*B, were predicted in all strains except ATCC 7061. *Fos*B opens the epoxide ring and destroys the activity of the antibiotic. Genes associated with resistance to β-Lactam antibiotics, such as carbapenem, cephalosporin and penams, were predicted as follows: *bc*I and *bc*II in 3 A ES; *bc*I and FIM-1 in 7 C ES; *bc*I, *bc*II, *Bla*, and SIM-1 in F60006; *bc*I and THIN-B in ATCC 49,063, and BPU-1 (class D) in ATCC 7061. Genes for resistance to peptide antibiotics such as colistin A or polymyxin E1 and colistin B or polymyxin E2 were also predicted: the mobile colistin resistance *mcr*-4.1 in 3 A ES, ATCCs 14,579 and 49,063, and *mcr*-4.2 in 7 C ES and 1 L BW. In contrast, strain F60006 harbors *bah*A, which encodes an enzyme inactivating bacitracin.

**Table 4:** Resistomes predicted by the resistance gene identifier (RGI) tool

Strains	Gene symbol	Drug class	AMR Gene Family
3 A ES, ATCC 14,579, ATCC 49,063	MCR-4.1	Peptide antibiotic; Colistin A, polymyxin E1	Mobile colistin resistance (MRC) phosphoethanolamine transferase
7 C ES	MCR-4.2	Colistin A, polymyxin E1; Colistin B, polymyxin E2	
	*ept*A	Peptide antibiotic	pmr phosphoethanolamine transferase
1 L BW	*ugd*		
F60006	*Bah*A	Peptide antibiotic	Bah amidohydrolase
3 A ES, 7 C ES, ATCC 14,579, ATCC 49,063	mdtG	Fosfomycin	Major facilitator superfamily (MFS) antibiotic efflux pump
3 A ES, 7 C ES, 1 L BW, ATCC 14,579, ATCC 49,063, F60006	FosB	Fosfomycin	Fosfomycin thiol transferase
3 A ES, 7 C ES, ATCC 14,579, ATCC 49,063	bcl	Cephalosporins, penams	Bc beta-lactamase
3 A ES, F60006	bcll		
7 C ES	TMB-1		Tripoli metallo beta-lactamase
	FIM-1		FIM metallo-beta-lactamase
ATCC 49,063	THIN-B	Carbapenem, cephalosporins, penams	THIN beta-lactamase
F60006	SIM-1		SIM beta-lactamase
	*bla*1	Penams	Class A *B. anthracis* Bla beta-lactamase
ATCC 7061	BPU-1		BPU beta-lactamase
3 A ES	*fus*C	Fusidic acid	Fusidic acid inactivation
1 L BW	*acr*R	Fluoroquinolone antibiotic; cephalosporin; glycylcycline; penam; tetracycline antibiotic; rifamycin antibiotic; phenicol antibiotic; triclosan	Resistance-nodulation-cell division (RND) antibiotic efflux pump
ATCC49063	*vga*ALC	Macrolide antibiotic; lincosamide antibiotic; streptogramin antibiotic; tetracycline antibiotic; oxazolidinone antibiotic; phenicol antibiotic; pleuromutilin antibiotic	ABC-F ATP-binding cassette ribosomal protection protein
F60006	*mup*B	Mupirocin	Antibiotic-resistant isoleucyl-tRNA synthetase (ileS)
ATCC 7061	BPU *cat*86	Phenicol antibiotic	Chloramphenicol acetyltransferase (CAT)

In addition to resistance genes shared across multiple strains, RGI also predicted two genes unique to individual strains in our study: *fusC*, fusidic acid resistance determinant, was only identified in 3 A ES and *vga*ALC, encoding an ABC-F subfamily protein conferring resistance to streptogramin A antibiotics and related compounds, was only predicted in ATCC 49,063.

**Fig. 1:**
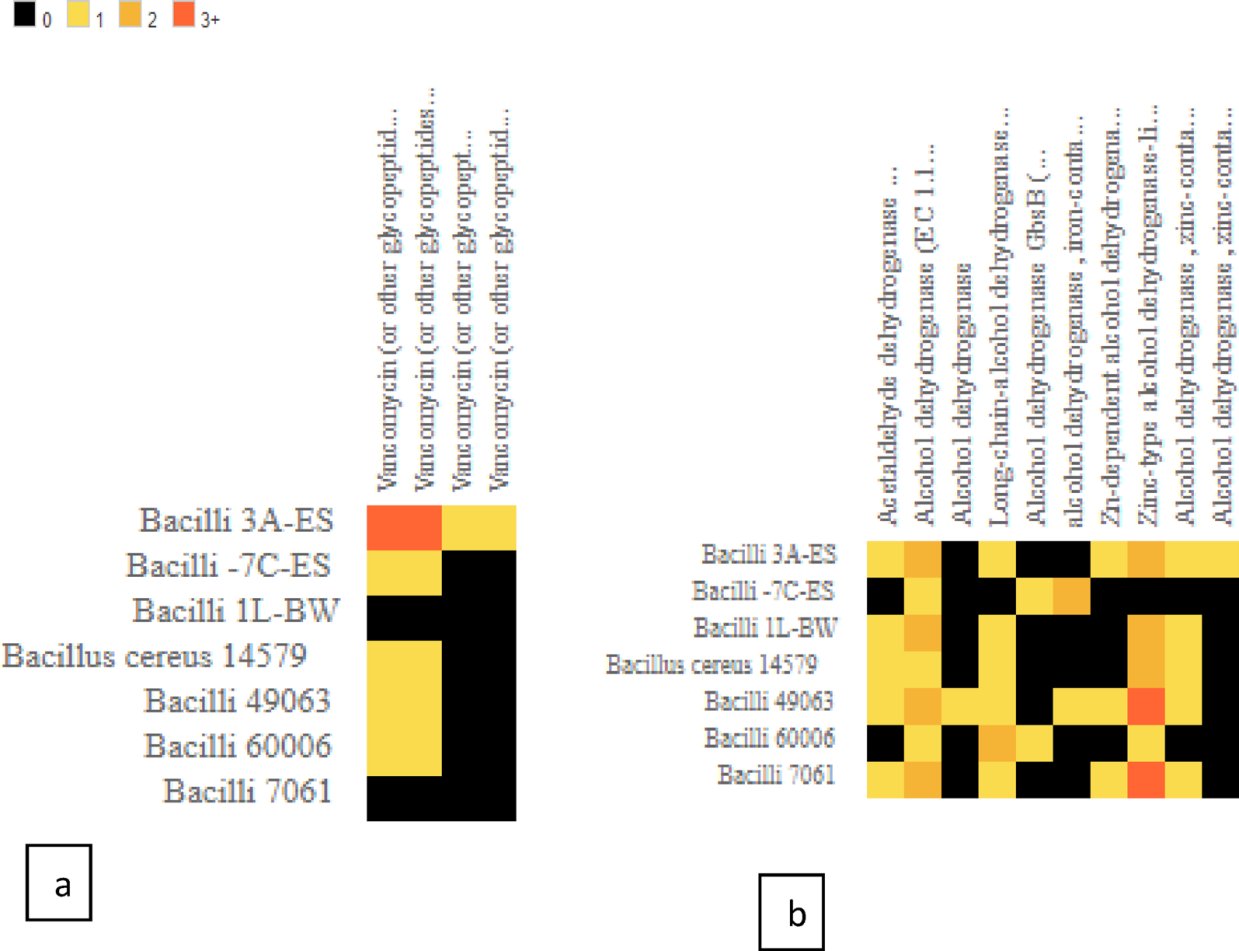
Heatmap predicted protein families of vancomycin (**a**) and alcohol dehydrogenase containing zinc (**b**) in 7 strains. Black color signifies no copy, yellow means one-copy, light orange means 2 copies, and dark orange means at least 3 copies

Additional predictions were observed by the heatmap protein comparison tool in BV-BRC: two types of vancomycin resistance determinants in 3 A ES: *van*F/M and *van*RB (Fig. 1a), and two copies of *adh*B (Fig. 1b), encoding an alcohol dehydrogenase containing zinc; *adh*B has been observed to have role in resistance to nitric oxide and oxidative stress [35].

### Phylogenetic tree comparison

**Fig. 2:**
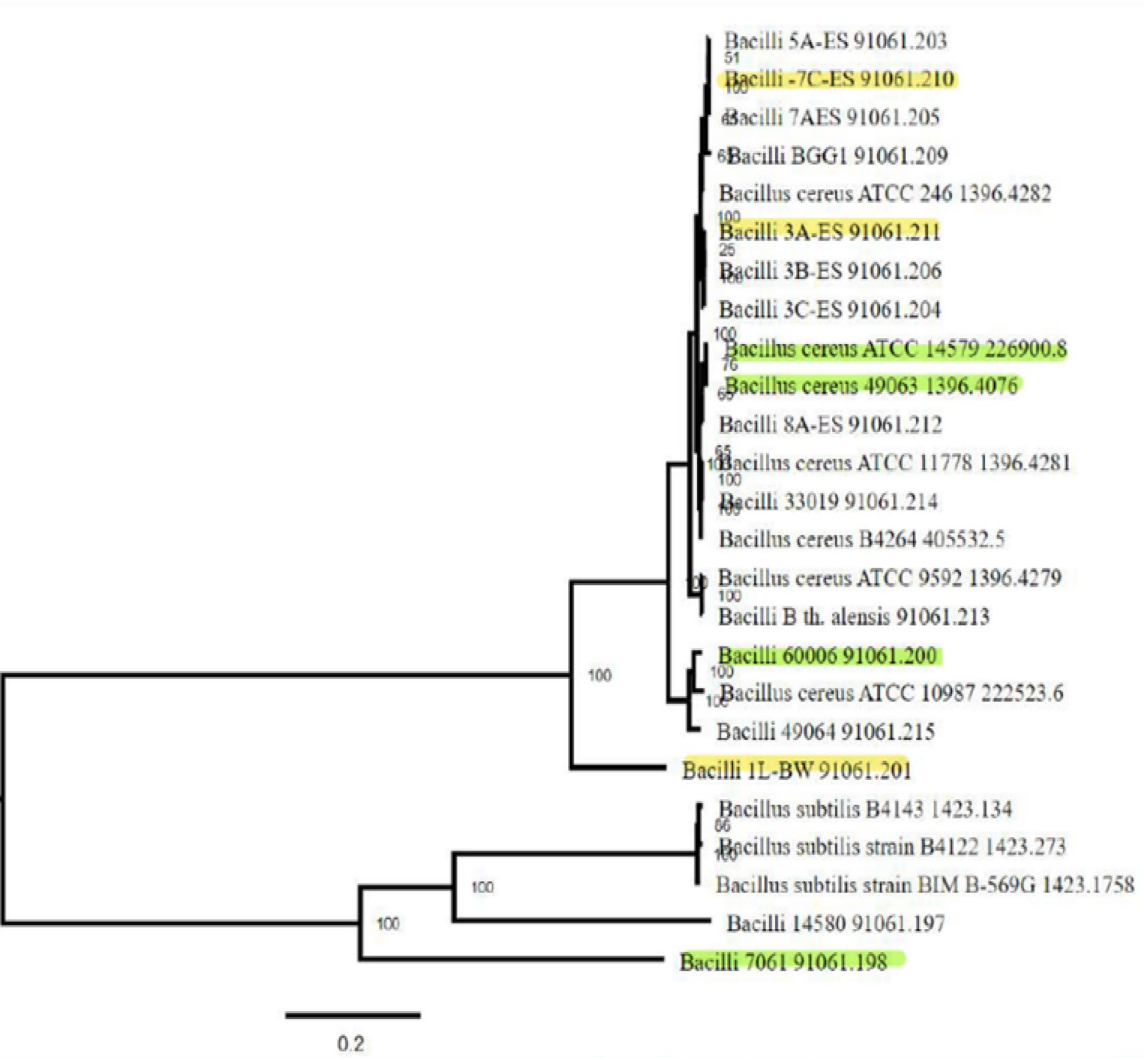
Phylogenetic tree constructed by BV-BRC using private, reference and representative *Bacillus* genomes. *B. cereus* strains 3 A ES, 7 C ES, 1 L BW, ATCC 14,579, ATCC 49,063, F60006, and *B pumilus* ATCC 7061 were used in this study. The cosmetic isolates are displayed in yellow and the other strains in this study are highlighted in green

The phylogenetic tree generated by BV-BRC from our set of twenty-five genomes showed that strains in our study panel were genomically diverse and that the isolates from cosmetics (displayed in yellow in Fig. 2) did not cluster together. As expected, the control strain ATCC 7061, previously established to be a *B. pumilus*, was well-separated from the *B. cereus* s.l. strains. Similar to the BTyper classement, our seven selected strains were grouped in three clusters, distributed across the tree among the representative *B. cereus* strains. Two eye shadow cosmetic isolates, 3 A ES and 7 C ES, and ATCC 14,579 and 49,063 were classified in Group IV, and clustered with most of the representative strains. Guinebretiere et al. [13] reported that the strains from this Group are *B. cereus sensu stricto* mesophilic strains growing in a temperature range of 10–45 °C and are frequently identified with clinical and food related adulterations and outbreaks. Similar to the previous Group, Group III strains in which F60006 was classified are mesophilic with a growth range from 15 to 45 °C. Group III is known to be the most diverse Group possessing strains with virulent plasmids coding for cereulide, anthrax virulent genes, *or insecticidal cry toxins* [36]. F60006, identified as *B. mosaicus*, was closely related on the tree with a representative food poisoning isolate, *B. cereus* ATCC 10,987 [37], which is reported to contain a similar plasmid as *B. anthracis* pXO1. Finally, the strain 1 L BW identified as *B. cytotoxicus* was the unique strain in Group VII. The strains in this Group are moderate thermotolerant with a growth range of 20 to 50 °C [13] and have been associated to rare but fatal cases of diarrheal disease [38]. Similar to the selected *B. cereus* strains that were allocated into a large clade composed of subclades, *B. pumilus* and the representative strains of *B. subtilus* and of *licheniformis* ATCC 14,580 were into the other single clade composed of a subclade indicating closeness among the strains.

### Protein family comparisons of selected strains

**Fig. 3:**
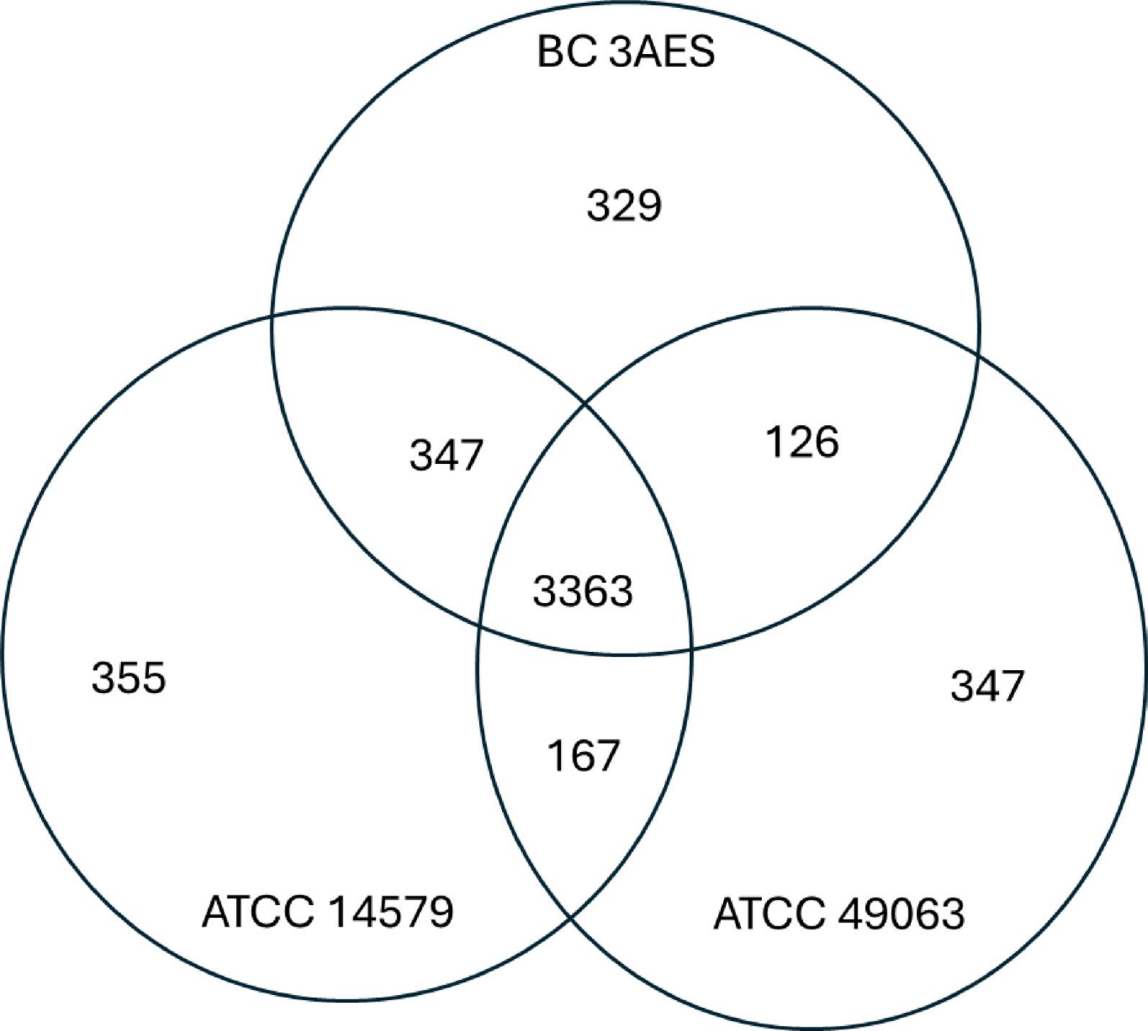
Comparison of protein families for three *B. cereus* s.l. strains. The protein families of each strain are represented by a circle. Protein families that are conserved across the three genomes, the core genome is at the center. The overlapping regions showed the number of protein families that match a specified function. The non-overlapping regions showed the number of conserved protein families or accessory genome, unique to the strain

The average growth/respiration of ATCC 14,579 and 49,063 were statistically similar to that of F60006 and 3 A ES; however, the average growth/respiration of 3 A ES was significantly different from F60006 as seen in Fig. 7. To explore this further, we compared the protein families in the three strains assigned to *B. cereus* s.l. Group IV. The resulting Venn diagram showed both the commonalities and diversity among these strains: the predicted core genome contained 3363 protein families, with 329 protein families found exclusively in 3 A ES, 347 in ATCC 49,063, and 355 in ATCC 14,579 (Fig. 3; see also Supplemental Material). The resilience of 3 A ES may be attributable to the enzymes, efflux pumps, transporter proteins, and transmembrane receptors predicted to be among those 329 protein families that were not found in the other selected strains.

### Phenotype Vitek^®^ 2 BCL sensitivity/resistance result

**Fig. 4:**
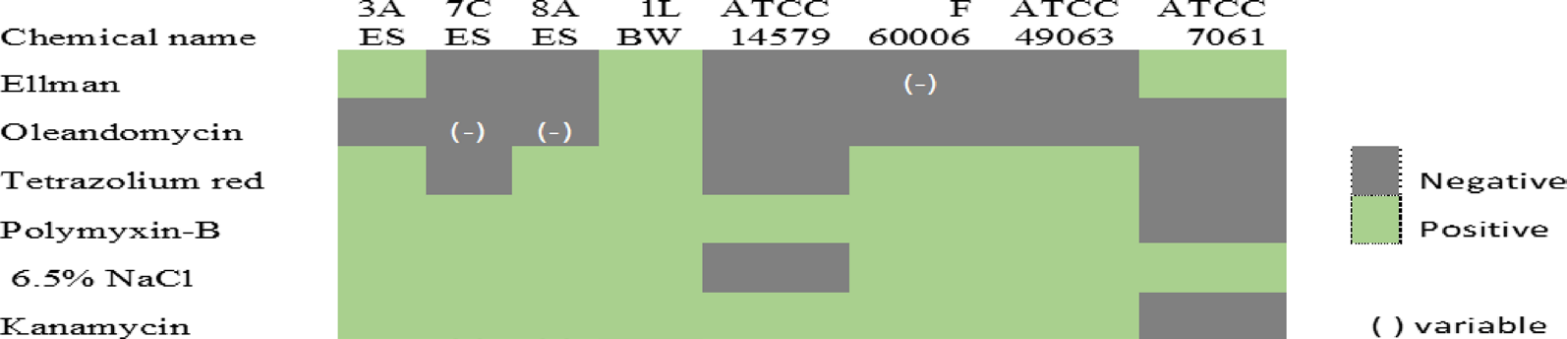
VITEK^®^ 4 sensitivity/resistance biochemical profiles of selected strains

Vitek BCL card identifies all the strains 3 A ES, 7 C ES, 1 L BW, ATCC 14,579, ATCC 49,063 and F60006 as *B. cereus* and suggests additional tests to detect the presence of crystal toxins and rhizoids within those strains. ATCC 7061 was identified as *B. pumilus*. This study focused on the sensitivity/resistance, so Fig. 4 shows the results from the Vitek 2 BCL card. Each strain exhibited a unique pattern of sensitivity and resistance. All the strains but ATCC 7061 were resistant to kanamycin (0.006 mg/well) and polymyxin B (0.00093 mg/well). There were also traits shared across multiple strains; for instance, all the strains except 1 L BW appeared sensitive to oleandomycin (0.003 mg/well), with weak evidence of resistance exhibited by cosmetic isolates 8 A ES and 7 C ES. The strains 3 A ES, 8 A ES, 1 L BW, F60006, and ATCC 49,063 were resistant to tetrazolium red (0.0189 mg/well) while 7 C ES and ATCC strains 14,579 and 7061 were not. Notably, two isolates from cosmetics, 3 A ES and 1 L BW, and the non-*cereus* strain ATCC 7061 were positive to the Ellman test reagent (0.03 mg per well); a weak reaction appeared in the well for F60006. This result implies 1Lthe presence of free thiol groups that have been associated with resistance to oxidative stress, as well as biofilm formation [39]. The only strain that could not grow in a solution of 6.5% NaCl (1.95 mg/well) was ATCC 14,579.

### Phenotype Microarray-Biolog (PM) results

#### PM sensitivity / resistance

Of the 240 compounds tested in the PM plates, six compounds were completely inhibitory to the growth of all the tested *B. cereus* strains: novobiocin, troleandomycin, tylosin tartrate, sodium metavanadate, sodium orthovanadate, and vancomycin. Similar to the results of other studies [1, 40], our isolates showed sensitivity to vancomycin. Interestingly, despite the predicted presence of *van*F/M and *van*RB by the heatmap prediction tool, 3 A ES remained as sensitive to vancomycin as the strains that did not carry those genes. Only seven compounds were completely inhibitory at all four concentrations to 3 A ES, with eight for ATCC 14,579, 11 for F60006, 12 for 8 A ES, 16 for 7 C ES, 13 for 1 L BW and ATCC 49,063, and 14 for ATCC 7061. Most of the strains were resistant to lactam, antimicrobial peptides, tetracyclines, macrolides, and phosphonic antibiotics, although sensitive to glycopeptides. Conversely to the results of other studies [1, 41–43] our isolates were resistant to amoxicillin, gentamicin, ciprofloxacin, chloramphenicol and doxycycline. In our study, the unique strain 7 C ES was susceptible to doxycycline, and other strains resistant, partially resistant or minimally resistant. Similarly, all the strains were fully resistant or resistant in PM 14 and resistant or partially resistant in PM 11.

**Fig. 5:**
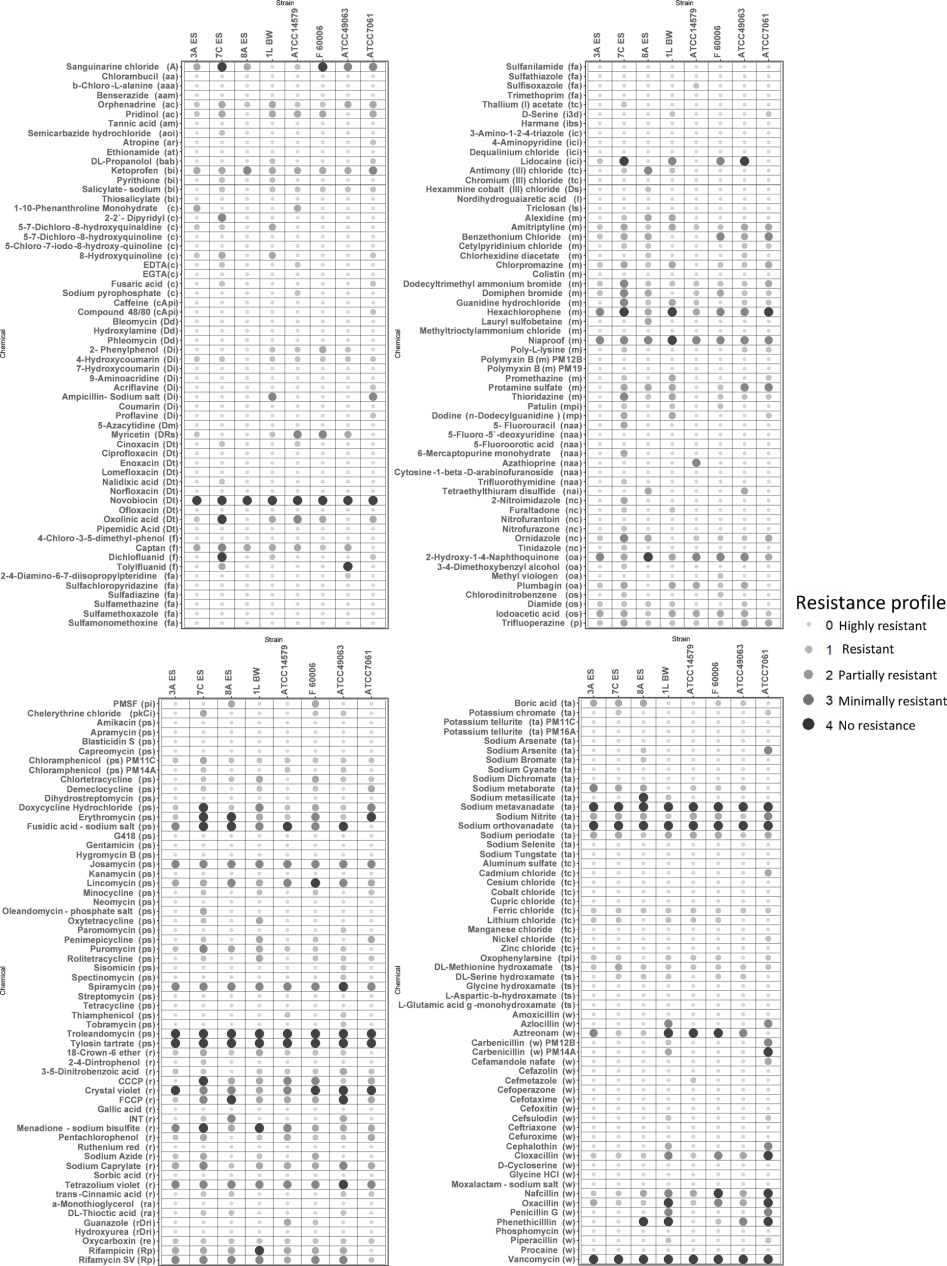
Sensitivity/resistance patterns of selected strains. Targets: protein synthesis inhibitors (ps; 35), cell wall-acting antibiotics (w; 27), membrane (m; 20), toxic anion (ta; 17), respiration (r; 16), toxic cation (tc; 13), folate antagonist (fa; 10), DNA topoisomerase (Dt; ), chelator (c; 10), DNA intercalator (Di; 8), nucleic acid analog (naa; 7), nitro compound (nc; 6), tRNA synthetase (ts; 5), biofilm inhibitor (bi; 4), oxidizing agent (oa; 4), fungicide (f; 4), ion channel inhibitor (ici; 3), DNA damage (Dd; 3), oxides sulfhydryls (os; 3), RNA polymerase (Rp; 2), ribonucleotide reductase inhibitor (rr; 2), cyclic AMP phosphodiesterase inhibitor (cAp; 2), tyrosine phosphatase (tp; 1), respiratory enzyme (re; 1), reducing agent (ra; 1), protein kinase C inhibitor (pkC; 1 compound), protease inhibitor (pi; 1), phenothiazine (p; 1), nucleic acid inhibitor (nai; 1), microtubulin polymerization inhibitor (mpi; 1), membrane permeability (mp; 1), lipoxygenase (l; 1 compound), lipid synthesis (ls; 1), catalase inhibitor (ci; 1), 3PGA dehydrogenase (3d; L-serine and pantothenate synthesis) inhibitor [1], imidazoline binding site (ibs; 1), DNA synthesis (Ds; 1), DNA methylation (Dm; 1), DNA and RNA synthesis (DRs; 1), beta-adrenergic (ba; 1), ATPase (A; 1), antituberculosis (at; 1), antimicrobial (am; 1), anticholinergic acid (aa; 1), amine oxidase inhibitor (aoi;1), alkylating agent (ala; 1 compound), acetyl choline receptor (acr; 1), Aa metabolism (Aam; 1), and Aa analog (Aaa; 1)

The results of the PM11-PM20 phenotypic tests are displayed in Fig. 5.

### PCA analysis of resistance phenotypes and growth patterns of the strains

**Fig. 6:**
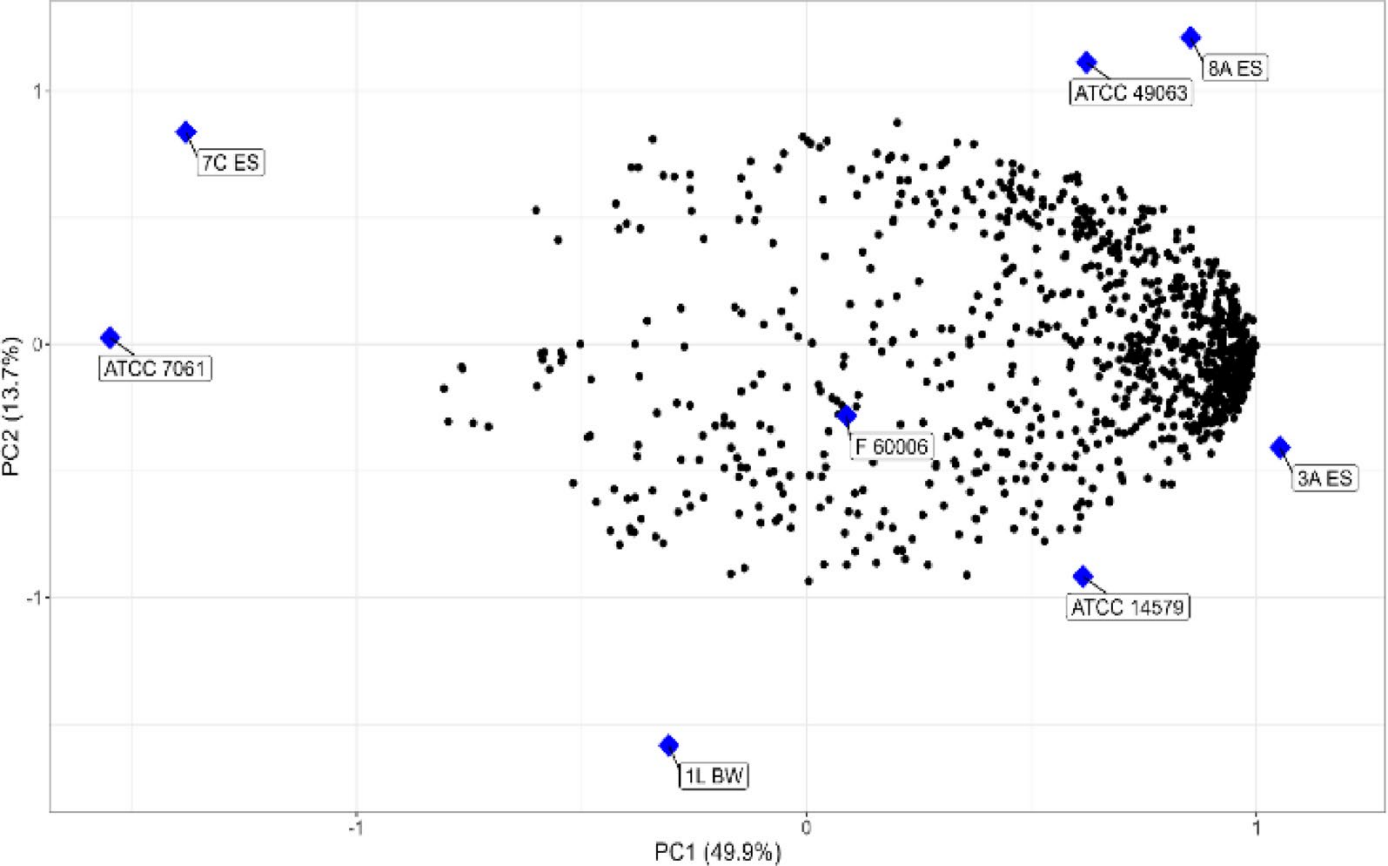
Principal component analysis of the phenotypic microarray profiles of the 8 strains of *Bacillus* that were obtained from analysis of 960 phenotypic tests (PM11-PM20). The strains in blue and the phenotypic tests in black dot were plotted in X-Y diagram

PCA was used to understand the relationship between the strains and the antimicrobials. These data were standardized beforehand, with results displayed in Fig. 6. The first principal component (PC1), displayed on the horizontal axis, captured 49.9% of phenotype variation and separated strains 7 C ES, 1 L BW and ATCC 7061 from 3 A ES, 8 A ES, ATCCs 14,579, 49,063, and F60006. The second principal component (PC2), displayed using the vertical axis, captured an additional 13.7% of phenotypic variation and provided good separation of strains 3 A ES, ATCC 14,579, and F60006 from strains 8 A ES and ATCC 49,063, and separated 1 L BW 7 C ES from ATCC 7061. These results indicate that strains 7 C ES, 1 L BW and ATCC 7061 are phenotypically different from 3 A ES, 8 A ES, F60006, as well as from ATCC strains 14,579 and 49,063. Moreover, the fact that they are grouped in two different homogenous groups based on PCA analysis confirmed the different patterns of genotypic and phenotypic resistance observed among these strains.

**Fig. 7:**
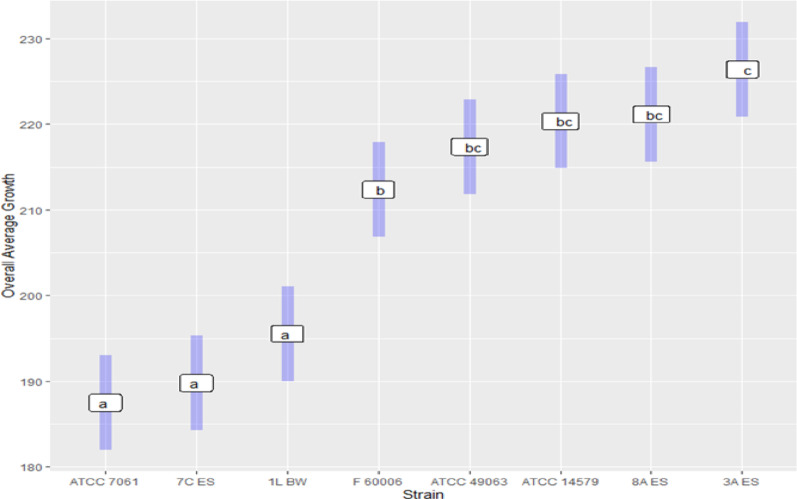
Tukey comparison of average growth/respiration by strains in PM wells. Groups sharing the same letter designation are not significantly different in their average growth/respiration performance, while groups with different letters are considered significantly different (*p* < 0.05)

Although the Tukey comparison of average growth/respiration responses (strain/sensitivity/ resistance) showed a significant difference (*p* < 0.001) among the strains, there were no differences identified between the growth of cosmetics isolates and the isolates obtained from other sources (*p* = 0.56). While ATCC 7061 was the least resistant among this set of strains, its growth was not statistically different from that of 7 C ES or 1 L BW. In addition, ATCC 7061 was out of 240 compound susceptible to 14. Besides the resistant genes and the concentration of the compounds, their resistance could also be due to the presence of ICEBs1-like element that contributes to bacterial resistance even in sensitive environments like spacecraft assembly facilities [44]. The strain demonstrating the highest growth response was 3 A ES, although that difference was only significant compared to the growth of F60006 (Fig. 7).

### Discrepancies between Vitek and PM analyses

The strains in this study harbored genes predicted to encode protection or repair proteins, stress/acid responses, efflux pumps, and other genetic determinants that should confer resistance to most of the compounds included in the PM arrays. However, the phenotypic analyses did not always confirm those resistances: there were some discrepancies between the VITEK^®^ 2 and PM results. For example, ATCC 7061 appeared sensitive to polymyxin and kanamycin only according to VITEK^®^ 2. Similarly, although ATCC 7061 grew in presence of polymyxin in the PM plates, in line with presence of *mcr*-4.1, *mcr*-4.2, *ept*A, or *ugd*, in its genome sequence (indicated by the RGI tool), it appeared sensitive to polymyxin according to VITEK^®^ 2. The VITEK^®^ 2 card may contain a higher concentration of polymyxin, overwhelming the inherent resistance mechanism of the strain. In contrast, the VITEK^®^ 2 showed 1 L BW as the only strain fully resistant to oleandomycin (0.003 mg/well), but the PM plates indicated that all eight strains were resistant to the undisclosed concentration(s) of oleandomycin phosphate salt used in those test wells. These discrepancies may be because not only oleandomycin phosphate salt is less potent than oleandomycin but also that VITEK had a higher concentration of the active ingredient than PM.

### Discrepancies between genotypic and phenotypic analyses

The predicted presence of *van*B-type in 3 A ES did not allow resistance to vancomycin, a glycopeptide, when tested in the PM assay. *van*A-type genes show resistance to vancomycin and teicoplanin while *van*B-type genes show suscepbility to teicoplanin. One of the explanation could be the strains and the concentrations, and the other explanation could be the lack of the ensemble of genes that contribute to the resistance of vancomycin [45]. Other studies showed that *B. cereus* group members, depending on the strains and concentrations, are intermediate resistant, resistant or resistant to vancomycin [46–49]. Likewise, the predicted presence of *fus*C in 3 A ES did not improve its resistance to over 1 L BW and F60006. It has been reported that mutations in the genes *fus*A are responsible for high-level resistance while the genes *fus*B, *fus* C, and *fus*D allow low-level resistance. Although its presence was not predicted in ATCC 7061, the strain was resistant to the compound suggesting other means of resistance to this compound such as the predicted presence of the elongation factor G. The predicted presence of the ATPases in the ATP-binding cassette F (ABFC) family, *vga*ALC genes, which confer resistance to macrolides, lincosamide, streptogramin, tetracycline, oxazolidinone, phenicol and pleuromutilin in ATCC 49,063, did not preclude the sensitivity to all the macrolides tested in the PM as tylosin, troleandomycin, spiramycin, and did not also allow as expected a full resistance to josamycin, erythromycin and chlortetracycline. The same, complete resistance of ATCC 49,063 to the aminoglycosides, paromomycin and tobramycin, and the lincosamides, lincomycin, was not achieved. The partial resistance of the strains except ATCC 49,063 and 14,579 to the phenicols, chloramphenicol in PM11 and the complete resistance of all the eight strains in PM 14 A showed not only the effect of the resistant genes, but also the contribution of the concentrations in the resistance of a strain, and it is confirmed by PM11 and PM14 where the concentration in PM11 (0.002–0.058 mM) was higher than in PM 14 (0.001–0.025 mM). Varied results were obtained with the β-lactams group despite the presence of beta-lactamase genes with ATCC 7061 followed by 1 L BW been the most susceptible strains to this group of antibiotics.

Overall, the presence of regulators plays a significant role in the resistance to the compounds of the *Bacillus*. Regulators such as PlcR / PapR, GntR, CodY control the expression of broad range virulence factors including phospholipases C, proteases, hemolysins, and enterotoxins and other genes are involved in adaptation to environment [50, 51]. The GntR family of transcriptional regulators were predicted in *B. cereus* 3 A ES, 7 C ES, ATCC 14,579, F60006, and in ATCC 49,063 and not in *B. cereus* 1 L BW and *B. pumilus* ATCC 7061. GntR family members are known to regulate various biological processes such as cell motility, glucose metabolism, bacterial resistance, and pathogenesis and virulence [52]. Another regulator, CodY that functions as GntR [53], was predicted in all tested isolates except in 7 C ES. All *B. cereus* strains including *B. pumilus* harbored LiaFSR, which function as part of the cell envelope stress response network and preserve the cell membrane integrity. LiaFSR with LiaR, also known as VraR, acts as a response regulator [54]. *B. pumilus* lacks the termination factor Rho, which is responsible for the suppression of pervasive, antisense transcription. It has been reported that Rho is involved in cell resistance to adversities [55]. On the other hand, ATCC 7061 was the unique strain in which transcription factors, FadR and TetR were predicted; they regulate diverse cellular processes including antibiotic resistance and metabolism [56]. In addition, BceR was predicted in ATCC 7061 genome. It is a response regulator protein involved in a two-component regulatory system that regulates diverse cellular processes including antimicrobial resistance, virulence, biofilm formation, and stress response to environmental changes [57]. The absence of GntR in 1 L BW and ATCC 7061, CodY in 7 C ES, and Rho in ATCC 7061 may be the reason of their weaknesses when compared to other strains.

## Conclusion

The difficulties of categorizing strains within the *B. cereus* group are well known [58]. The initial goal of this research was to describe the selected isolates from cosmetics to the isolates from the non-cosmetics. Our analysis determined that the isolates from cosmetics and non-cosmetics were genotypically diversified, resulting in diverse phenotypic sensitivity/ resistance patterns, including multidrug resistance. Nonetheless, strains obtained from cosmetics did not exhibit genomic features that would clearly distinguish them from the non-cosmetics, and our PCA analysis did confirm those findings. A large sample size may have allowed a better comparison of cosmetics to the non-cosmetic isolates.

The patterns of sensitivity we observed in response to specific environmental challenges in the Vitek and PM assays did not always match the predicted resistances from genomic analyses, which could be due in part to differences in the concentrations of the challenge substances used in each. Among the strains, 3 A ES remained the most resistant and could be added to a type collection given its distinctive resistance profile.

It is also important to note that both the Vitek and PM tests can only evaluate actively metabolizing cells; any spores present might still be capable of germinating at some future time if the environment became favorable to growth. The wide range of environmental niches acceptable to *B. cereus* group members provide many opportunities for bacterial survival [59]. For example, spores of ATTC 49,063 have been shown to survive in disinfectant solutions used in cleaning food preparation surfaces [60]. Further research is needed to investigate these possibilities. In addition, studies including more cosmetic isolates, transcriptomic confirmation, and survival of the strains in cosmetic products should be undertaken.

## Supplementary Information

Below is the link to the electronic supplementary material.

Supplementary Material 1

Supplementary Material 2

Supplementary Material 3

## Data Availability

The datasets generated in this study have been included in the study. Raw data are available from the corresponding author without making an undue reservation to any qualified researcher. The whole genome sequencing data in this study have been deposited in GenBank under Bio Project: PRJNA574468, PRJNA574468, PRJNA574468.

## Abbreviations

ATCC: American Type Culture Collection
BUG agar: Biolog Universal Growth
BV-BRC – PATRIC: Bacterial and Viral Bioinformatics Resource Center - Pathosystems Resource Integration Center
BW: Baby Wipes
CARD: Comprehensive Antibiotic Resistance Database
ES: Eye Shadow
MLST: Multi-Locus Sequence Typing
*pan*C: Pantoate-Beta Alanine Ligase C
PCA: Principal Components Analysis
PGFams: Global Protein Families
PM: Phenotype Microarrays™
RGI: Resistant Gene Identifier
SNP: Single Nucleotide Polymorphism
